# Perceived stress levels among patients treated for neovascular age-related macular degeneration with anti-VEGF injections

**DOI:** 10.1007/s00417-025-06883-w

**Published:** 2025-06-28

**Authors:** Ida Nilsson, Hugo Senra, Karthikeyan Baskaran, Camilla Mohlin, Antonio Filipe Macedo

**Affiliations:** 1 Department of Medicine and Optometry, Linnaeus University, Kalmar, Sweden; 2 Department of Ophthalmology, Region Kalmar Län, Kalmar, Sweden; 3 Institute of Electronics and Informatics Engineering of Aveiro (IEETA), University of Aveiro, Aveiro, Portugal; 4 School of Health and Social Care, University of Essex, Colchester, U.K.; 5 Department of Chemistry and Biomedical Sciences, Linnaeus University, Kalmar, Sweden; 6 Center of Physics-Optometry and Vision Science, University of Minho, Braga, Portugal; 7 Norra Kajplan 6, Hus Vita, Kalmar, 391 82 Sweden

**Keywords:** Neovascular age-related macular degeneration, Vision loss, Perceived stress, Perceived social support

## Abstract

**Purpose:**

The aim of this study was to assess perceived stress levels in patients with nAMD undergoing treatment with anti-VEGF injections, and to investigate psychosocial and visual factors that can be associated with perceived stress among these patients.

**Methods:**

We recruited 202 patients diagnosed with nAMD (mean age of 78 years) who had received three or more anti-VEGF injections and had been scheduled for further treatments. To measure perceived stress, participants completed the Perceived Stress Scale-10 (PSS10). For the associated factors, the participants also completed the National Eye Institute Visual Function Questionnaire-25 and Multidimensional Perceived Social Support. Participants completed the questionnaires at home before an upcoming treatment scheduled at the hospital. Best corrected visual acuity was measured at the hospital before the treatment. Factors associated with PSS10 scores were examined using multiple regression models.

**Results:**

Participants with near vision impairment perceived higher stress levels than those without near vision impairment (*p* = 0.034). Younger age (β = -0.15, *p* = 0.003), better visual acuity (β = -4.20, *p* = 0.036), poorer perceived social support (β = -1.21, *p* < 0.001), and poorer self-reported visual function (β = -0.16, *p* < 0.001) were significantly associated with increased levels of perceived stress.

**Conclusions:**

Our study highlighted factors potentially associated with increased perceived stress in nAMD patients undergoing anti-VEGF treatment. Self-reported visual function, in particular near-vision, and perceived social support are factors that can be addressed to reduce the levels of stress and risk of mental health disorders in this patient group.

**Supplementary Information:**

The online version contains supplementary material available at 10.1007/s00417-025-06883-w.

## Key messages

*****What is known***:**

- Neovascular AMD (nAMD) is perceived by patients as a threat to their vision and mental health.

*****What is new***:**

- Perceived stress among patients who are receiving intravitreal anti-VEGF injections is correlated with perceived vision performance but not with clinically measured visual acuity.
- Perceived social support protects against stress among patients with nAMD receiving anti-VEGF treatments.
- Improving satisfaction with individual vision performance, in particular at near, might help patients with nAMD receiving anti-VEGF treatments to meet their visual demands and reduce their stress.

**Supplementary Information:**

The online version contains supplementary material available at 10.1007/s00417-025-06883-w.

## Introduction

Age-related macular degeneration (AMD) is a leading cause of irreversible vision loss and blindness worldwide [1, 2]. The neovascular type of AMD (nAMD) can be treated with intravitreal injections carrying anti-vascular endothelial growth factors (anti-VEGFs). This treatment can preserve the retinal morphology and prevent vision loss [3–5]. Patients under treatment often regard frequent hospital visits as time-consuming and costly, with some patients feeling that the information given to them about the disease is too brief and some get dissatisfied with treatment results [6]. In general, patients treated with anti-VEGF injections are likely to experience anxiety and emotional distress in relation to treatment administration and treatment outcomes [7–9].

Previous studies suggested that nAMD patients are more likely to show symptoms of anxiety and depression than comparable groups of the population without the disease [10–13]. These symptoms enhance the detrimental impact of nAMD on quality of life and the burden of regular medical treatments, leading to a loop of emotional distress [8, 14]. The association between anxiety symptomatology and nAMD has been described in previous research [8, 14, 15]. However, there is still very limited knowledge on to what extent patients might also experience high levels of stress, and which factors can explain perceived stress among these patients.

The aim of the current study was to assess perceived stress in patients with nAMD undergoing treatment with anti-VEGF injections (irrespective of anti-VEGF agent), and to determine psychosocial and visual factors associated with perceived stress in those patients. From our previous research, we hypothesize that stress levels are influenced by perceived social support and reduced near visual acuity [15].

## Methods

The research protocol was approved by the Swedish Ethics Review Authority (Dnr 2020/04083) and was conducted in accordance with the tenets of the Declaration of Helsinki. The study was conducted at the Department of Ophthalmology in the regional hospital in Kalmar, Sweden. Patients were consecutively invited between March 2021 and March 2023. Invitation to participate in the study was sent out to patients diagnosed with “degeneration of macula and posterior pole (ICD 10 Diagnosis Code H353)” scheduled for treatment with intravitreal anti-VEGF injection. Inclusion criteria: (1) patients in the clinic that were actively receiving anti-VEGF treatments after completing the “loading phase” (consisting of 3 injections in monthly intervals), (2) patients under treat-and-extend regimen (treatment interval is based on treatment response and regularly assessed). This was to ensure that all participants were in a chronic stage of their disease to attenuate the potential effect of an initial acute psychological impact of the diagnosis, the fear of the injections or both [16]. Exclusion criteria: patients with a definite or suspected diagnosis of mental disorders that could impair their ability to provide informed consent, e.g., dementia. All patients in the sample were treated with bevacizumab (25 mg/mL) in the loading phase. The treating physician switched some to ranibizumab (10 mg/mL) or aflibercept (40 mg/mL) when hers or his medical assessment considered the efficacy of the treatment with bevacizumab below expected.

### Measures

#### Perceived stress

Perceived stress was measured with the Perceived Stress Scale 10 (PSS10). The scale measures the experience of stressful events and is sensitive to chronic stress [17]. In this scale, respondents are asked to estimate how often life felt unpredictable, uncontrollable, and overloaded in the last month. The possible answers are: “Never = 0 points”, “Almost Never = 1 point”, “Sometimes = 2 points”, “Fairly often = 3 points” and “Very often = 4 points”. Questions are asked using expressions of vulnerability (negative items 1, 2, 3, 6, 9 & 10), e.g., *“In the last month*,* how often have you been upset because of something that happened unexpectedly?”* or expressions of invulnerability (positive items 4, 5, 7 & 8), e.g., *“In the last month*,* how often have you felt confident about your ability to handle your personal problems?*”. Negative items are scored 0 to 4 and form the “emotional distress” subscale. Positive items are re-scored in reverse order 4 to 0 and form the “coping-ability” subscale. The maximum score is 40 points. Higher scores indicate more perceived stress [17]. The scale also exists in 14-item and 4-item versions; but they are less reliable than the 10-item version [18, 19]. The scale has been widely used in clinical research, across different medical conditions, and have previously been validated in studies conducted in patients with AMD [20] and other ophthalmic conditions [21–24]. Participants completed the PSS10 at home before the scheduled treatment visit.

#### Vision-targeted health status

The National Eye Institute Visual Function Questionnaire (VFQ-25) was developed by the National Eye Institute (NEI) in the USA to measure how visual disabilities or symptoms influence health-related quality of life in patients with chronic eye diseases [25]. The instrument assesses individuals’ vision-targeted health status, including vision-related quality of life (VRQoL). The 25-item scale covers vision-related health domains: general health, general vision, distance activities, near activities, colour vision, peripheral vision, driving, ocular pain, role limitations, social function, dependency, mental health, and expectations for future vision. All answered items are averaged together when calculating the score. The maximum total score for the scale is 100 points. Each individual domain can also be scored up to 100 points. Higher scores indicate better visual function. VRQoL measured by the VFQ-25 was used in this study as a “non-clinical” or “self-reported” visual function measure. Participants completed the VFQ-25 at home before the scheduled treatment visit.

#### Perceived social support

The Multidimensional Perceived Social Support (MSPSS) Scale has been extensively used to measure perceived social support [26]. MSPSS is formed by 12-items, scored in a 7-point scale ranging from “very strongly disagree” (1 point) to “very strongly agree” (7 points). All answered items are averaged together when calculating the total score. The maximum score is 7 points. Higher scores indicate higher perceived social support. The scale can be used to measure “total” perceived social support or specifically: “family” (items 3, 4, 8, and 11), “friends” (items 6, 7, 9, and 12) and “significant other” (items 1, 2, 5 and 10). Participants completed the MSPSS at home before the scheduled treatment visit.

#### Demographic and clinical data

Participants reported financial conditions as: very good, good, bad, or very bad, and education levels as: academic degree, high school diploma, elementary school diploma, or below elementary school level. Participants answered all questions at home before the scheduled treatment visit.

The spectrum of comorbidities in the sample was diverse and we decided to make a summary using a known comorbidities index– the Charlson Comorbidity Index (CCI). The CCI can be derived from electronic records with disease codes, and we used the same procedure that has been adopted for medical research in Sweden [27]. The CCI is a predictor of 1-year mortality risk from comorbid diseases in which the conditions considered are weighted in their seriousness between 1 and 6, index indicate higher risk of mortality. The maximum weighted score in the Swedish protocol is 30 [27]. Age can also be accounted for in CCI. The age groups and their weights are as follows: <50 years (0), 50–59 years (1), 60–69 years (2), 70–79 years (3), and ≥ 80 years (4) [28]. When adding weights for age to the comorbid conditions the maximum score is 34. The same electronic medical records used to compute CCI were used to compute the number of participants with appointments related to depression or anxiety.

#### Visual acuity

Participants’ refractive error was measured with an autorefractometer and confirmed subjectively with trial lenses. Visual acuity (VA) was measured with the best possible correction and, therefore, values in the manuscript correspond to best corrected VA. Refractive errors are not reported here because records were inconsistent. Monocular VA was measured at distance and near in both eyes. VA in the better-seeing eye was used for analysis. Distance VA was measured in a dark room using the logarithmic Early Treatment Diabetic Retinopathy (ETDRS) back-lit illuminated chart (Precision Vision). The test was performed at either 1–2 m which enabled measurement of VA between 1.7 and 0.0 logMAR. Near VA was measured using the LIX reading chart, which contains texts within the range of 4-to-24-point size. The near VA test was performed in a well-lit room with an extra reading light at 33 cm with the addition of + 3.00D to account for the reading distance. When participants were unable to read any letters in the ETDRS chart at 1 m, near VA was not tested.

For some analyses, distance VA in logMAR units was used to categorize patients’ level of vision loss according to the World Health Organization classification of visual impairment (VI) [29]. The categories were: normal vision (VA ≤ 0.3 logMAR); mild visual impairment (0.3 < VA ≤ 0.5 logMAR), moderate or severe (including blindness) visual impairment (VA > 0.5 logMAR).

#### Data analysis

We used multiple imputations with the mice package (R Statistics) to complete missing data in the PSS10 [30]. The package performs multiple imputations of missing values using the “polr” function, a proportional odds logistic regression method. This method is suitable for imputing missing values in ordinal response variables. Data was then analyzed with SPSS (v.21, Chicago, USA) and R Statistics. The normality of the distribution of the variables was tested using the Kolmogorov-Smirnov test, Shapiro-Wilk, or both. We report median and inter-quartile range (IQR) for non-parametric outcomes and mean and standard deviation (SD) for parametric outcomes. We used non-parametric tests such as Spearman Rho and Mann-Whitney U for univariate analysis, when the variables failed the normality tests. To determine the factors associated to perceived stress we used multivariate regression analysis. All statistical assumptions for linear regression models were met, including normal distribution of residuals (Shapiro-Wilk test with p-values > 0.05), residuals not auto-correlated (Durbin-Watson test with p-values > 0.05), heteroscedasticity not present (Breusch-Pagan Test with p-values > 0.05), and VIF values not suggesting multicollinearity (values ranging from 1.0 to 1.5). For all tests, statistical significance was set to *p* < 0.05.

## Results

A total of 202 patients were recruited. The median age was 78 years (56–98 years), 133 (66%) were female and 99 (34%) were male. The median number of injections received (total for both eyes) was 14 (IQR: 7–25), and the median time since the first injection was 18 months (IQR: 6–52). There was a small, albeit significant, correlation between number of injections and distance VA (rho = 0.19, *p* = 0.008), indicating that patients with more injections tended to have worse VA in their better seeing eye.

Three participants failed to answer all items in the PSS10, four in the MSPSS, and one in the VFQ-25; these were excluded from further analysis. Information was missing regarding financial status in 37 (18%), education level in 38 (19%), and comorbidities in 5 (2%) participants. One participant was not able to read 24 points on the near VA chart, and distance VA in logMAR was converted to N-points as a proxy to near VA.

Thirty-one (19%) participants reported very good financial status, 122 (74%) reported good, 9 (6%) reported bad, and 3 (2%) reported very bad financial status. Forty-three (26%) participants had an academic degree, 19 (11%) had a high school diploma, 62 (37%) had an elementary school diploma, and 42 (25%) had an education level below elementary school.

For the 199 answers available for analysis, the median PSS10 score was 12.00 (IQR: 7.00–17.00) points out of 40 points. The median score for the “emotional distress” subscale was 7.00 (IQR 5.00–11.00) out of 24 points. The median for the “coping-ability” subscale was 4.00 (IQR: 2.00–7.00) out of 16 points.

The median CCI excluding weights for age was 1.00 (IQR 0.00–2.00), and when adding weights for age the median CCI was 4.00 (IQR: 3.00–5.00). Thirty participants (15%) had at least one hospital episode related to depression, and 21 (11%) had episodes related to anxiety. Of these, 10 (5%) participants experienced episodes of both depression and anxiety. The PSS10 score was higher among participants with episodes of depression compared to the rest of the sample, the difference between medians was 4 (Mann-Whitney U-test, Z=−2.63, *p* = 0.009). The score for the “emotional distress” subscale score was higher amongst participants with episodes of depression compared to the rest of the sample, the difference between medians was 4 (Mann-Whitney U-test, Z=−2.61, *p* = 0.009). The effect of having episodes of depression, anxiety, or both was no longer significant in the multivariate regression models described below.

In the better-seeing eye, the median VA at distance was 0.18 (IQR: 0.06 − 0.42) logMAR, and at near was 4 (IQR: 4.00–8.00) points. Participants were categorized based on VA in their better-seeing eye according to the WHO classification of visual impairment (VI) [29]. The categories and the distribution of the participants are summarized in Table 1. No participant was blind in both eyes, but 51 (25%) were blind in one eye.

**Table 1:** Classification of the participants visual impairment (VI) according to the international classification of diseases, eleventh revision (ICD-11), world health organization (WHO) 2019/2021and based on the better seeing eye

Vision categories	VA	Number of participants (%)
Normal distance vision	≤ 0.3 logMAR	133 (66)
Mild VI	> 0.3 to ≤ 0.5 logMAR	33 (16)
Moderate VI	> 0.5 to ≤ 1.0 logMAR	35 (17)
Severe VI	> 1.0 to ≤ 1.3 logMAR	1 (1)
Blindness	> 1.3 logMAR	0 (0)
Near VI	> 6 N-points	63 (31)

The median score for the MSPSS was 6.08 (IQR: 5.17 - 6.75) points out of 7 points. The median score for the “family” subscale was 6.50 (IQR: 5.25 -7.00), “friends” subscale was 5.50 (IQR: 4.25-6.50), and for the “significant other” subscale was 6.50 (IQR: 5.50-7.00), each has a maximum score of 7 points. As summarized in Table 2, reduced scores on perceived social support were associated with increased levels of perceived stress, emotional distress and lower levels of coping ability. The strongest correlation with PSS10 was the “family” subscale, while the weakest was the “significant other” subscale.

**Table 2:** Correlations between the perceived stress (PSS10) and its subscales (emotional distress and coping-ability) and multidimensional perceived social support (MSPSS) and its subscales (family, friends and significant other), visual function questionnaire-25 (VFQ-25) and its subscales (distance activities and near activities) and Charlson comorbidity index (CCI), and visual acuity (VA) at distance and at near

	PSS10	Emotional distress	Coping-ability
Perceived social support			
Total score (MSPSS)	*rho*=−0.363 *p* < 0.001**	*rho*=−0.281 *p* < 0.001**	*rho*=−0.340 *p* < 0.001**
Family (MSPSS)	*rho*=−0.405 *p* < 0.001**	*rho*=−0.323 *p* < 0.001**	*rho*=−0.357 *p* < 0.001**
Friends (MSPSS)	*rho*=−0.334 *p* < 0.001**	*rho*=−0.273 *p* < 0.001**	*rho*=−0.302 *p* < 0.001**
Significant other (MSPSS)	*rho*=−0.244 *p* < 0.001**	*rho*=−0.159 *p* = 0.026*	*rho*=−0.262 *p <* 0.001**
Comorbidities			
Total score (CCI)	*rho* = 0.005 *p* = 0.921	*rho*=−0.044 *p* = 0.543	*rho* = 0.044 *p* = 0.547
Visual function			
VRQoL (VFQ-25)	*rho*=−0.379 *p* < 0.001**	*rho*=−0.372 *p* < 0.001**	*rho*=−0.292 *p* < 0.001**
Distance activities (VFQ-25)	*rho*=−0.298 *p* < 0.001**	*rho*=−0.299 *p* < 0.001**	*rho*=−0.218 *p* < 0.001**
Distance VA (LogMAR)	*rho* = 0.053 *p* = 0.454	*rho* = 0.001 *p* = 0.986	*rho* = 0.078 *p* = 0.275
Near activities (VFQ-25)	*rho*=−0.250 *p* < 0.001**	*rho*=−0.238 *p* < 0.001**	*rho*=−0.188 *p <* 0.001**
Near VA (N-points)	*rho* = 0.117 *p* = 0.099	*rho* = 0.083 *p* = 0.246	*rho* = 0.101 *p* = 0.155

*Indicates a statistically significant test result for a p-value equal or more than 0.01 but less than 0.05, and **indicates a statistical significant test result for a *p*-value less than 0.001

The median VFQ-25 score was 77.57 (IQR: 61.19 - 87.31) points out of 100 points. The median score for visual function for “distance activities” was 75.00 (IQR: 50.00- 87.50), and for “near activities” was 58.33 (IQR: 50.00 - 83.33 ), each has a maximum score of 100 points. Non-clinical visual function measures given by the VFQ-25 (Table 2) were correlated with measures of perceived stress. Reduced non-clinical visual function was associated with increased levels of perceived stress and emotional distress as well as lower levels of coping ability. Correlations between clinically measured VA and PSS10, emotional distress and coping-ability were not statistically significant.

### Comparison between vision categories and multivariate regression analysis

Table 3 summarizes group comparisons. We compared PSS10 scores between different categories: (1) normal vision vs. any VI, (2) normal near vision vs. near VI, and (3) none vs. one blind eye. We also divided the participants based on their VRQoL measured with the VFQ-25: (4) good to excellent (score ≥ 80) vs. fair to very poor (score < 80).

**Table 3:** Median scores and inter-quartile range (IQR) for perceived stress-(PSS10), emotional distress and coping-ability

	PSS10		Emotional distress		Coping-ability	
	*Median (IQR)* *Range 0–40*	*Mann Whitney-U*	*Median (IQR)* *Range 0–24*	*Mann Whitney-U*	*Median (IQR)* *Range 0–16*	*Mann Whitney-U*
Normal distance VA (*n* = 133)	12 (17.0–7.0)	*U* = 4912 *z =* 1.106 *p =* 0.269	7 (11.0–5.0)	*U* = 4510 *z =* 0.065 *p =* 0.948	4 (6.5-2.0)	*U* = 5114.5 *z =* 1.636 *p =* 0.102
Any distance VI (*n* = 69)	14 (18.5-8.0)		7 (12.0–4.0)		5 (7.0–3.0)	
Normal near VA (*n* = 139)	12 (16.0–7.0)	*U* = 5082 *z =* 2.115 *p =* 0.034*	7 (10.0–5.0)	*U* = 4936.5 *z =* 1.731 *p =* 0.083	4 (7.0–2.0)	*U* = 4819 *z =* 1.423 *p =* 0.155
Near VI (*n* = 63)	15 (20.0–8.0)		8 (13.0–4.0		5 (7.0–3.0)	
No eye blind (*n* = 151)	12 (17.0–7.0)	*U* = 4180 *z =* 1.146 *p =* 0.252	7 (11.0–4.0)	*U* = 3788 *z =* 0.040 *p =* 0.968	4 (6.0–2.0)	*U* = 4350.5 *z =* 1.633 *p =* 0.102
One eye blind (*n* = 51)	14 (18.0–8.0)		7 (11.0–5.0)		6 (8.0-0.20)	
Excellent/Good VRQoL (*n* = 88)	10 (15.0–5.0)	*U* = 6734.5 *z =* 4.770 *p <* 0.001**	6 (9.0–4.0)	*U* = 6689.5 *z =* 4.662 *p <* 0.001**	3 (6.0–1.0)	*U* = 6331 *z =* 3.773 *p <* 0.001**
Fair/Very poor VRQoL (*n* = 113)	15 (19.0–11.0)		9 (12.0–6.0)		5 (7.0–3.0)	

Values are given for vision categories based on vision category and VRQoL

*Indicates a statistically significant test result for a *p*-value equal or more than 0.01 but less than 0.05, and **indicates a statistically significant test result for a *p*-value less than 0.001

Table 4 summarizes two multivariate regression models with PSS10 scores as the dependent variable (outcome measure). The difference between Model 1 and Model 2 is how VA was used. Univariate statistics is given in the supplemental results file. For Model 1, we use VA in the better-seeing eye as a continuous independent predictor. For Model 2, we converted VA into vision categories with the cut-offs given in methods and in Table 2.

**Table 4:** Multivariate regression analysis of predictors of perceived stress-(PSS10): model 1 and model 2

	Model 1			
	*Estimates*	*std. Error*	*CI*	*p*
Age (Years)	-0.15	0.05	-0.25– -0.04	**0.006**
Gender: Female*	-0.19	0.82	-1.80 – 1.43	0.821
VA better-seeing eye (logMAR)	-4.20	1.98	-8.11– -0.28	**0.036**
MSPSS (Total score)	-1.21	0.30	-1.80– -0.62	**<0.001**
VRQoL (VFQ-25)	-0.16	0.03	-0.22– -0.11	**<0.001**
Observations	194			
R^2^ / R^2^ adjusted	0.260 / 0.240			
AIC	1208.622			
*Reference category “Male”				
	Model 2			
	*Estimates*	*std. Error*	*CI*	*p*
Age (Years)	-0.16	0.05	-0.27– -0.06	**0.003**
Gender: Female	-0.13	0.82	-1.75– 1.49	0.879
Mild Visual Impairment*	0.22	1.13	-2.00– 2.44	0.843
Moderate to Severe Visual* Impairment*	-2.60	1.16	-4.89– -0.30	**0.027**
MSPSS (Total score)	-1.19	0.30	-1.79– -0.60	**<0.001**
VRQoL (VFQ-25)	-0.16	0.03	-0.21– -0.11	**<0.001**
Observations	194			
R^2^ / R^2^ adjusted	0.266 / 0.242			
AIC	1209.029			
*Reference category “No visual impairment” defined by the better-seeing eye				

The multivariate regression Model 1 indicates that age, VA (LogMAR), perceived social support (MSPSS), and VRQoL (VFQ-25) were predictors of participants perceived stress (PSS10). Younger age (β = −0.15, ρ = 0.003), better VA (β = −4.20, ρ = 0.036), poorer perceived social support (β = −1.21, ρ < 0.001), and poorer VRQoL (β = −0.16, ρ < 0.001) were associated with greater levels of perceived stress.

Model 2 is consistent with Model 1, participants with moderate to severe visual impairment showed significantly lower perceived stress levels than participants with no visual impairment or mild visual impairment (β = −2.60, ρ = 0.027). This subdivision into VI categories provides a better picture of what can be expected when VA falls into a standard classification of VI. We considered this helpful to generate a clearer message because a change of 1 logMAR unit of VA (Model 1) is a large step from normal vision to blindness.

## Discussion

With the current study we wanted to characterize perceived stress levels in patients with nAMD undergoing treatment with anti-VEGF injections. The results suggest that VA, VRQoL, perceived social support, and age are independently associated with perceived stress levels. These factors associated with perceived stress levels are discussed from two different perspectives: whether factors can be modified; and whether clinicians should pay special attention to certain patient sub-groups when the factors cannot be modified.

Visual acuity (VA) was associated with PSS10 scores. In our multivariate regression analysis, the first model indicates that a loss of one line of letters in the vision chart corresponds to an estimated reduction of 0.42 points in PSS10 scores. The second model confirmed that participants with moderate to severe visual impairment reported less perceived stress than those with normal vision or mild visual impairment. A possible explanation for these results is that patients with better vision may have a more recent diagnosis and therefore are more likely to have a less developed coping strategy to adapt to vision changes as well as be afraid of going blind in the future [8, 14, 15]. In contrast, patients in a more advanced chronic stage are more likely to have developed an effective coping strategy to deal with changes in vision, which also explains why vision loss and mental health might not have a direct association [12, 14, 31]. This is also suggestive of that VA can be a proxy for disease duration, which is partially corroborated by our results, as a correlation between the number of injections and distance VA was found. In line with this, previous research has highlighted the presence of anticipatory anxiety of going blind in patients with a recent onset of nAMD who are at the beginning of anti-VEGF treatment [7, 8, 46]. Future larger-scale studies will clarify the link between anxiety and nAMD, nevertheless these patients might benefit from regular screening for anxiety and stress symptomatology, to help improving the adjustment to the disease and medical outcomes.

A previous similar study measuring perceived stress levels with the PSS10 in patients with AMD failed to find a correlation with VA [20]. The sample included 137 patients with all subtypes of AMD, some of whom were undergoing treatment with anti-VEGF injections. In studies using the PSS10 with other ophthalmic conditions, such as non-infectious uveitis (*n* = 120) [21] and primary open-angle glaucoma (*n* = 67) [22], a negative correlation with VA, visual field loss or both was found. However, in both studies the multivariate analysis failed to find significant associations between VA and perceived stress levels. In our study, only the multivariate models suggested a significant association between VA and perceived stress levels. This might be explained by the type of patient group examined in our study, but also by the fact that our sample is larger than the ones included in previous similar studies [29–31].

In this study, VRQoL given by the VFQ-25 was independently associated to PSS10 scores with higher VRQoL associated with lower perceived stress levels. The VFQ-25 assesses several domains (subscales) that include, among others, questions about perceived visual function at distance and near as well as well-being or emotional distress [25, 32]. It is, therefore, intuitive and consistent to find an association between VFQ-25 scores and PSS10.

Our univariate analysis revealed an association between perceived visual function for distance and near and PSS10 scores (Table 2). A previous study has shown that higher perceived stress levels can predict future and persistent self-reported vision and hearing problems [33]. The authors speculate that this prediction is bi-directional– as has been proven to be true for self-reported visual function and depression [34, 35]. The extent to which reduced visual function affects patients’ lifestyle and autonomy may depend on individual expectations of vision performance and strategies to compensate for vision loss [36]. Low vision rehabilitation can improve patients’ vision performance and help them to meet their expectations [36, 37]. Therefore, we consider perceived visual function a modifiable factor to buffer perceived stress levels. Vision rehabilitation is a suitable solution for patients who feel their vision is inadequate for achieving their goals, and may lead to better mental health and improved quality of life [14, 38].

The negative association found between levels of perceived social support and perceived stress is consistent with our previous research conducted in patients with AMD or diabetic retinopathy [15]. Patients with AMD are, in general, at risk of facing social isolation, which might be a result of different factors such as coping with vision loss, depression, lack of effective social support, and need for adjustments to enable an carry out everyday life activities without help [39–42].A good and effective perceived social support can enhance psychological resilience to stress [43] and therefore, it is a modifiable factor to reduce perceived stress levels among patients with nAMD.

In our study, perceived stress levels significantly decreased with age. This finding aligns with a study that characterized perceived stress among patients with dry eye [24]. Results of the current study and the dry eye study suggest that younger patients are more susceptible to higher levels of perceived stress than older patients [24]. This finding is also consistent with studies with the general population showing that perceived stress levels decrease, for example, after retirement [18, 19, 44, 45]. In summary, older age can be a protective factor against perceived stress in patients with nAMD.

The current study suggests potential benefits of screening patients with nAMD for perceived stress as part of the medical follow-up protocol, particularly in the early stages of the disease. Patients with high levels of stress can be referred to specialized services (e.g., vision rehabilitation, psychology, or social services), which might have a positive impact on nAMD treatment outcomes.

### Strengths and limitations

The current study provides a characterization of perceived stress levels in patients with nAMD, which may represent a prodromal stage of long-term symptoms of anxiety and depression [10, 46, 47]. Nevertheless, our findings are limited by the cross-sectional design of the study. Chronic stress (as measured by the PSS10) can be a consequence of a disease but also a contributing factor. In our study, we conceptualized symptoms of stress as “a consequence” of the disease. Evidence on the actual effect that psychological stress may have on measurable physical symptoms and treatment outcomes among patients with nAMD is still scarce, and more studies are needed to address this question.

## Conclusion

Our findings revealed that VA, VRQoL, perceived social support, and age are independent predictors of perceived stress in patients with nAMD who are under treatment with anti-VEGF injections. Self-reported visual function, in particular near-vision, and perceived social support are modifiable factors that can be addressed to reduce the risk of future anxiety and depression disorders in this patient group.

## Electronic supplementary material

Below is the link to the electronic supplementary material.

Supplementary Material 1 (DOCX 70.8 KB)
