# Supplementary figures and images for: Perceived stress levels among patients treated for neovascular age-related macular degeneration with anti-VEGF injections
Source: Graefes Arch Clin Exp Ophthalmol. 2025 Jun 28;263(9):2523–31. doi: 10.1007/s00417-025-06883-w (PMC12513873; doi:10.1007/s00417-025-06883-w)

**UNIVARIATE STATISTICS FOR VARIABLES USED IN MODELS 1 AND 2**


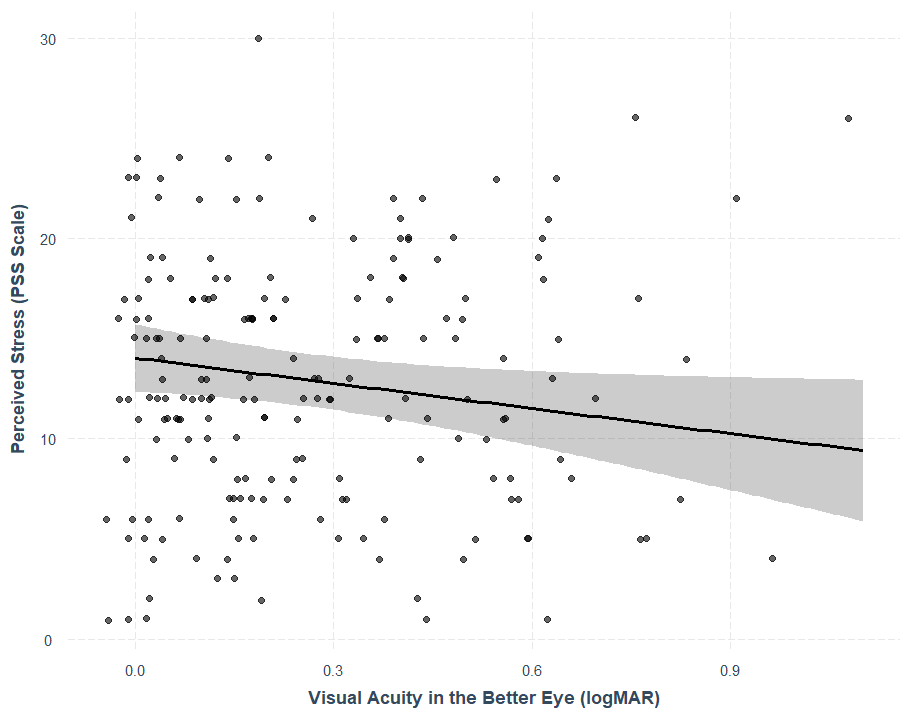


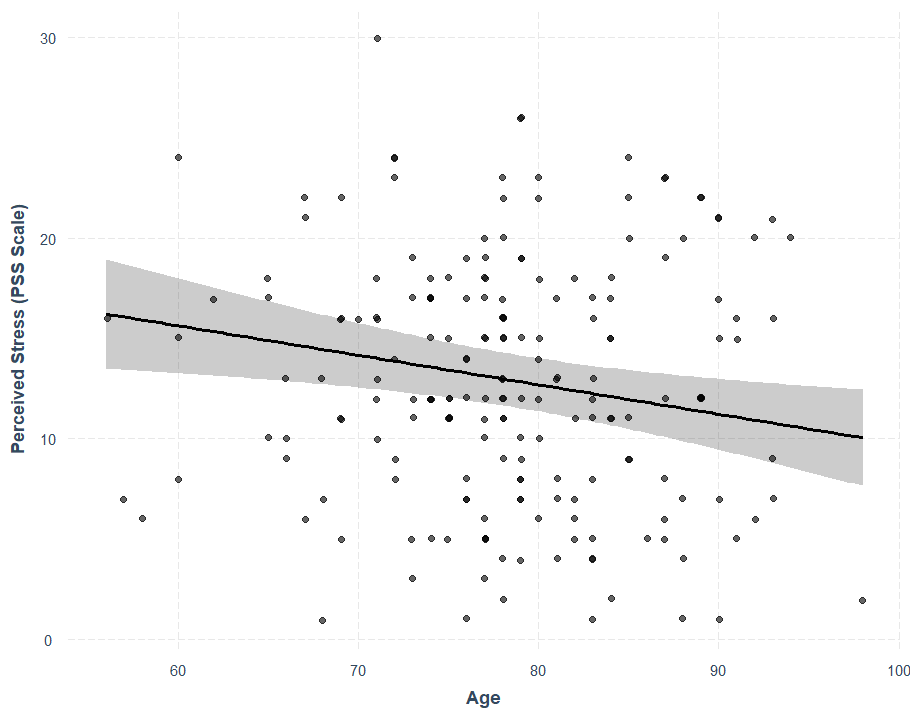


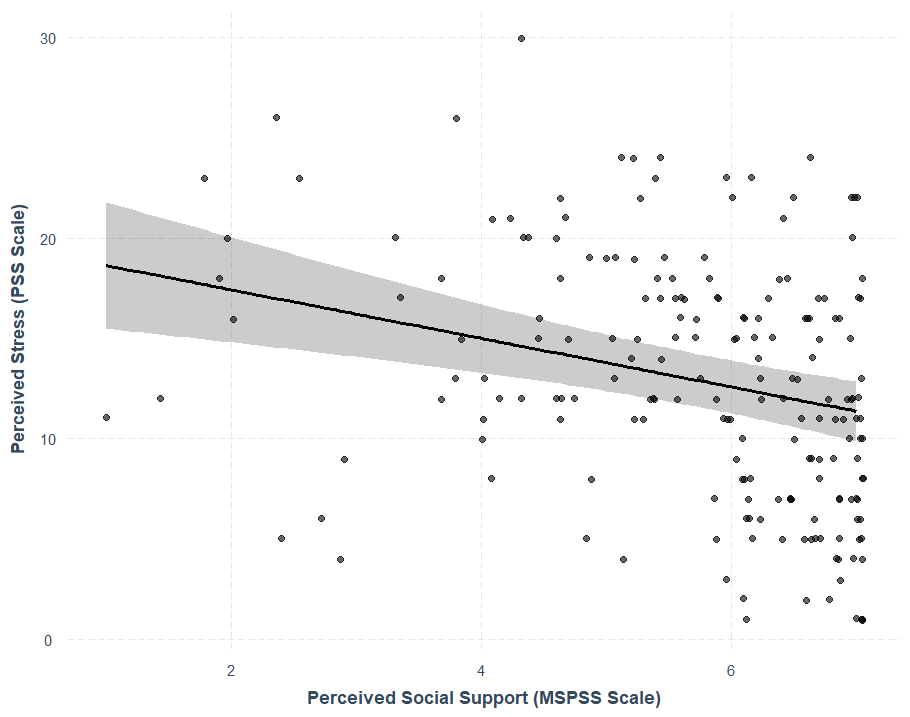


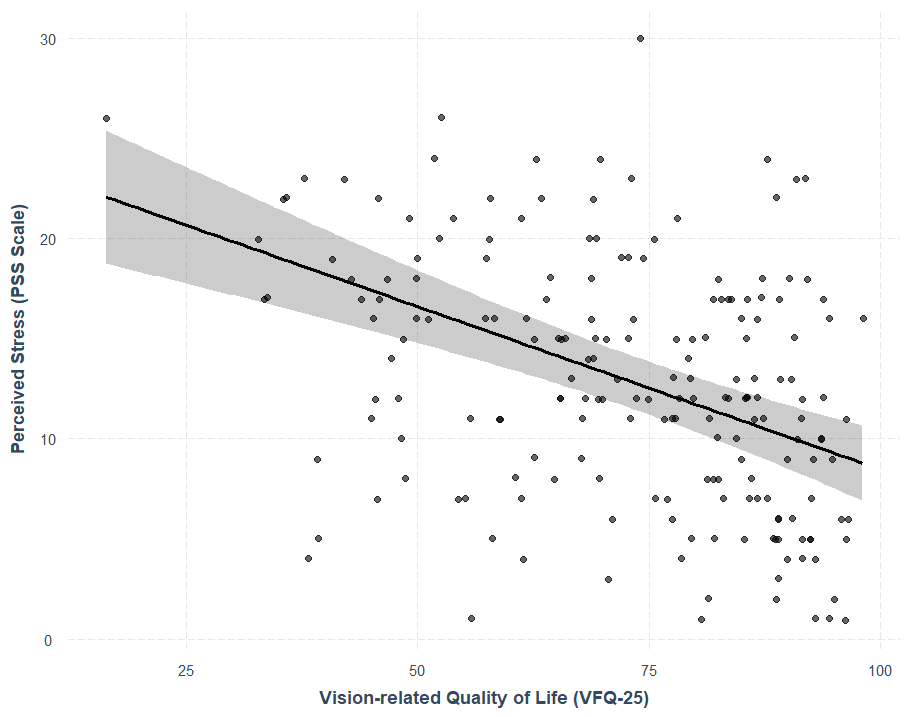

Supplement: Supplementary file 1 — Supplementary Material 1 (DOCX 70.8 KB) [file 417_2025_6883_MOESM1_ESM.docx]
